# Acute retinol mobilization by retinol-binding protein 4 in mouse liver induces fibroblast growth factor 21 expression

**DOI:** 10.1016/j.jlr.2022.100268

**Published:** 2022-08-27

**Authors:** Julia S. Steinhoff, Carina Wagner, Ulrike Taschler, Sascha Wulff, Marie F. Kiefer, Konstantin M. Petricek, Sylvia J. Wowro, Moritz Oster, Roberto E. Flores, Na Yang, Chen Li, Yueming Meng, Manuela Sommerfeld, Stefan Weger, Andrea Henze, Jens Raila, Achim Lass, Michael Schupp

**Affiliations:** 1 Charité Universitätsmedizin Berlin, corporate member of Freie Universität Berlin and Humboldt-Universität zu Berlin, Institute of Pharmacology, Berlin, Germany; 2 Institute of Molecular Biosciences, NAWI Graz, University of Graz, Graz, Austria; 3 Charité Universitätsmedizin Berlin, corporate member of Freie Universität Berlin and Humboldt-Universität zu Berlin, Institute of Virology, Campus Benjamin Franklin, Berlin, Germany; 4 Martin Luther University Halle-Wittenberg, Institute of Agricultural and Nutritional Sciences, Halle, Germany; 5 Junior Research Group ProAID, Institute of Nutritional Science, University of Potsdam, Nuthetal, Germany; 6 Department of Physiology and Pathophysiology, Institute of Nutritional Science, University of Potsdam, Nuthetal, Germany; 7 BioTechMed-Graz, Graz, Austria

**Keywords:** vitamin A, retinoids, glucose, hepatic retinol mobilization, retinyl ester storage, obesity, hepatocyte, retinoid homeostasis, insulin resistance, atRA, all-*trans* RA, FGF21, fibroblast growth factor 21, HSC, hepatic stellate cell, ingWAT, inguinal WAT, NEFA, nonesterified fatty acid, pgWAT, perigonadal WAT, RA, retinoic acid, RAR, retinoic acid receptor, RARE, RA-response element, RBP4, retinol-binding protein 4, STRA6, stimulated by retinoic acid 6, UCP1, uncoupling protein 1, WAT, white adipose tissue

## Abstract

Hepatocytes secrete retinol-binding protein 4 (RBP4) into circulation, thereby mobilizing vitamin A from the liver to provide retinol for extrahepatic tissues. Obesity and insulin resistance are associated with elevated RBP4 levels in the blood. However, in a previous study, we observed that chronically increased RBP4 by forced *Rbp4* expression in the liver does not impair glucose homeostasis in mice. Here, we investigated the effects of an acute mobilization of hepatic vitamin A stores by hepatic overexpression of RBP4 in mice. We show that hepatic retinol mobilization decreases body fat content and enhances fat turnover. Mechanistically, we found that acute retinol mobilization increases hepatic expression and serum levels of fibroblast growth factor 21 (FGF21), which is regulated by retinol mobilization and retinoic acid in primary hepatocytes. Moreover, we provide evidence that the insulin-sensitizing effect of FGF21 is associated with organ-specific adaptations in retinoid homeostasis. Taken together, our findings identify a novel crosstalk between retinoid homeostasis and FGF21 in mice with acute RBP4-mediated retinol mobilization from the liver.

Vitamin A (retinol) and its derivatives (retinoids) regulate many processes fundamental for life (1, 2, 3). Apart from the visual cycle, most retinoid actions are due to binding of the retinol oxidation product retinoic acid (RA) and related compounds to nuclear retinoic acid receptor (RAR) and retinoid X receptors that function as ligand-activated transcription factors (4).

About 70% of retinoids in the body are located in the liver, whereas within the liver, 90–95% of retinoids are stored as retinyl esters in specialized hepatic stellate cells (HSCs) (5). Mobilization of hepatic retinoids requires hydrolysis of HSC-stored retinyl esters and transfer of retinol to hepatocytes, where it binds retinol-binding protein 4 (RBP4, also known simply as RBP) and is secreted into the blood stream, altogether a process incompletely understood (6). RBP4, a 21 kDa protein of the lipocalin family and highly expressed in hepatocytes, is the only specific transport protein for retinol in the circulation. Mice lacking RBP4 cannot mobilize hepatic retinoid stores, show signs of extrahepatic retinoid deficiency such as a visual impairment, and are highly dependent on dietary vitamin A intake (7, 8, 9, 10).

Besides hepatic retinol mobilization as its canonical function, RBP4 attracted significant interest in the context of metabolic diseases (11). Circulating RBP4 levels are increased in obese and insulin-resistant mice and humans (12), coinciding with elevated *Rbp4* mRNA expression in white adipose tissue (WAT) (13, 14), suggesting that RBP4 also acts as an adipokine (15). Moreover, injection of recombinant RBP4 or its transgenic overexpression in mice impaired glucose tolerance and induced insulin resistance (12, 16). Mechanistically, these effects were shown to involve elevated gluconeogenic gene expression in liver (12), the activation of a RBP4 receptor, stimulated by retinoic acid 6 (STRA6)-mediated Janus kinase 2/signal transducer and activator of transcription 5 signaling cascade (16), and a local inflammatory response in adipose tissue (17, 18, 19, 20) as RBP4-triggered events that lead to insulin resistance (21). However, several studies could not reproduce these findings (22, 23, 24), generating some controversy on whether RBP4 is indeed causative for developing insulin resistance (11). Although a consensus has not been reached, recent advances were made by showing that an adipocyte-restricted transgenic expression of human RBP4 did not elevate circulating RBP4 levels in mice fed normal chow diet, suggesting that it is not an adipokine in the classical sense. It was shown that adipocyte-expressed RBP4 may affect metabolic health by local effects in adipose tissue that, subsequently, drive perturbations in other organs like liver (25). Moreover and supporting the notion that RBP4 is not an adipokine, circulating RBP4 derives exclusively from hepatocytes since the transport protein was undetectable in serum of mice with hepatocyte-selective deletion of *Rbp4* (26).

Previously, we reported that a chronic elevation of hepatocyte-derived RBP4 in the circulation, which was quantitatively comparable to those observed in obese, insulin-resistant mice, did not impair glucose tolerance, irrespective of feeding normal chow or a high-fat diet (27). In another mouse model, we showed that an acute overexpression of hepatic RBP4 resulted in a robust retinol mobilization and induction of RAR target genes in extrahepatic cells such as in the stroma-vascular fraction of perigonadal WAT (pgWAT) (28). Thus, liver-derived RBP4 mobilizes hepatic retinoid stores, induces RAR-mediated gene expression in extrahepatic tissues, but even in the long term, does not impair glucose tolerance in mice.

Here, we show that an acute hepatic retinol mobilization induced by liver-specific RBP4 overexpression in mice reduces body fat content. This was associated with elevated concentrations of NEFA and β-hydroxybutyrate and increased hepatic expression and serum levels of fibroblast growth factor 21 (FGF21), a known inducer of fat turnover and insulin sensitivity. We found that retinol mobilization increases *Fgf21* expression of hepatocyte autonomously via RAR, and that FGF21 itself affects retinoid homeostasis in a tissue-specific manner. Hence, elevated RBP4 in the context of acute retinol mobilization, in contrast to the general perception of RBP4, improves metabolic control in mice, which we show to involve a novel crosstalk between hepatic retinol mobilization and FGF21. These findings have important implications for our understanding of the metabolic function of RBP4 and its link to retinoid homeostasis.

## Materials and methods

### Cloning, production, and purification of adenoviruses and adeno-associated viruses

Generation of adenoviruses expressing GFP or murine RBP4 under the control of a cytomegalovirus promoter is described elsewhere (28). Adenoviruses were purified from infected human embryonic kidney 293 cells by standard cesium chloride gradients and titers determined using the Adeno-X rapid titer kit (Clontech). Coral GFP or murine FGF21 complementary DNAs were amplified by PCR and cloned downstream of a Kozak sequence into the pds-adeno-associated virus (AAV) plasmids using *in-fusion* cloning (Clontech). Vectors were used to assemble self-complementary AAV vectors, driven by the synthetic and highly hepatocyte-specific LP1 promoter (29). AAVs of the 2/8 serotype (AAV2rep, AAV8cap) were generated as described previously (27). Purified AAVs were dialyzed against 0.9% NaCl and titers determined by quantitative PCR, amplifying a common LP1 promoter fragment (supplemental Table S1).

### Mouse studies and virus injections

Animal procedures were approved by the corresponding authorities in Berlin/Germany. Sample size estimates were performed using G∗Power (30). All mice were on a C57BL/6J genetic background, housed under standard 12 h light/12 h dark cycles, and fed normal chow diet (R/M-Haltung [sniff], 9% kcal fat, and 25 IE vitamin A/g before autoclaving). Mice, tail vein-injected with adenoviruses expressing GFP and RBP4, are described elsewhere (28). For ectopic expression of FGF21, equal titers of AAV2/8 (2E10 genomic copies) were injected via the tail vein into adult mice as previously described (27).

### Determination of serum and liver retinoids

Serum retinol and retinol and retinyl esters in tissues were analyzed by HPLC-photodiode array as described previously (28).

### Metabolic characterization

Body composition was analyzed by NMR (Minispec LF50; Bruker). Glucose tolerance was determined after 16 h of fasting and the intraperitoneal injection of glucose (2 g/kg) and repeated blood glucose measurements from the tail vein using glucose test strips (Contour Next; Bayer). Serum insulin was measured by ELISA (Rat Insulin ELISA; Crystal Chem). Serum NEFA and triglycerides were analyzed biochemically using commercial NEFA-HR (2) (FUJIFILM Wako Chemicals Europe) and triglycerides FS (DiaSys Diagnostic Systems) kits, respectively. Serum β-hydroxybutyrate was analyzed by test strips (FreeStyle precision pro; Abbott). Serum FGF21 was determined by ELISA (Quantikine ELISA mouse/rat FGF-21; R&D Systems). Food intake was determined in single-housed mice for 24 h.

### Gene expression

Spin columns (Qiagen or Macherey-Nagel) were used for RNA purification. Complementary DNA synthesis and quantitative PCR were performed as previously described (31). Gene expression was evaluated using standard curves while *36b4* or *Hprt* served as housekeepers. All primers are listed in supplemental Table S1.

### Immunoblotting

Protein isolation and quantification and immunoblotting using SDS-PAGE or SDS-free native conditions were performed as described previously (28). Primary antibodies are listed in supplemental Table S2, and horseradish peroxidase-conjugated secondary antibodies were used for detection (Thermo Fisher Scientific). Densitometric analyses were performed by ImageJ (32).

### Primary hepatocyte culture and treatments

Primary hepatocyte isolation was performed as previously described (33). Hepatocytes were cultured in DMEM for up to 96 h. Adenoviral infection was carried out overnight. Hepatocytes were transfected with an RA-response element (RARE)-containing luciferase reporter using Lipofectamine 2000 (Thermo Fisher Scientific) as described (34). Firefly luciferase reporter activity was normalized to cotransfected renilla luciferase (Dual Luciferase Reporter Assay; Promega). Treatment with all-*trans* RA (atRA) was done for 24 h at a concentration of 100 nM.

### Statistics

Data are presented as individual data points and mean ± SEM. Representative results of at least three independent cell culture experiments are shown. Mouse cohorts were analyzed using two-tailed Student’s *t-*tests or ANOVA as appropriate, and *P* < 0.05 was deemed significant.

## Results

### Acute retinol mobilization from liver induces fat turnover and insulin sensitization

**Figure 1:**
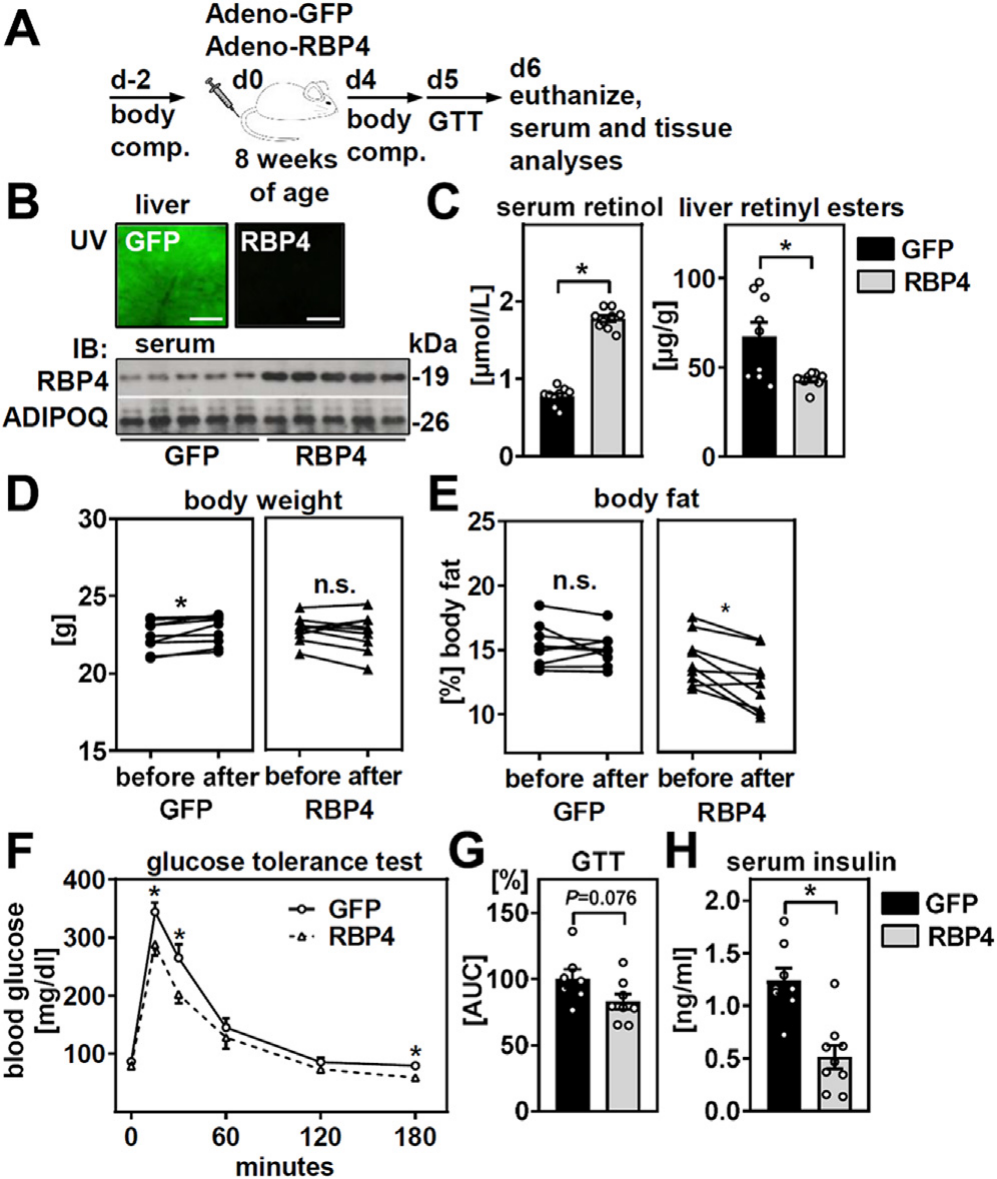
Acute retinol mobilization from liver induces fat turnover. Male C57BL/6J mice were tail vein-injected with adenoviruses encoding GFP or RBP4 and characterized as depicted in (A). B: Mouse liver was analyzed by UV microscopy, scale bars represent 200 μm, and serum RBP4 by immunoblotting. Adiponectin (ADIPOQ) served as loading control. C: Liver retinol and retinyl esters were quantified by HPLC. D: Body weights and (E) fat contents were determined by NMR before and after RBP4-mediated retinol mobilization. F: After an overnight fast, mice were injected intraperitoneally with glucose and blood glucose measured after indicated time points. G: Area under the curve (AUC) of glucose tolerance tests shown in (F). H: Serum insulin was measured by ELISA. Data are represented as individual data points and/or mean ± SEM and ∗*P* < 0.05 versus control mice expressing GFP.

Male C57BL/6J mice were tail vein-injected with adenoviruses expressing cytomegalovirus promoter-driven GFP or murine RBP4 and metabolically characterized within 6 days (Fig. 1A). We chose adenoviruses for their strong and fast transgene expression in liver within hours. Liver-specific RBP4 overexpression in these mice was reported previously (28, 34). Adenoviral infection induced strong GFP expression in livers of control mice and a robust increase in circulating RBP4 protein in mice receiving adenovirus encoding *Rbp4* (Fig. 1B, top and bottom panels). Correspondingly and as reported previously (28), serum retinol concentrations were increased by more than 2-fold and accompanied by a ∼35% reduction in hepatic retinyl ester levels, comprising quantification of retinyl palmitate, oleate, and stearate that account for >95% of all hepatic retinyl esters (35) (Fig. 1C). Hence, forced expression and secretion of RBP4 in mouse liver elicits an acute and robust mobilization of more than a third of all hepatic retinoid stores within only 6 days.

We then analyzed changes in body weights and fat content during the intervention. Body weights of control mice slightly increased, whereas in mice with liver-specific RBP4, overexpression did not change (Fig. 1D). Interestingly, relative body fat content declined only in mice with liver-specific RBP4 overexpression but not in control mice (Fig. 1E). Glucose tolerance was not impaired in mice with liver-specific RBP4 overexpression after an intraperitoneal glucose load (Fig. 1F), and the calculated areas under the curve were comparable (Fig. 1G). Moreover, serum insulin concentrations were lower in ad libitum-fed mice with liver-specific RBP4 overexpression (Fig. 1H). Hence, we conclude that acute retinol mobilization reduces body fat content and does not impair glucose tolerance.

### Acute retinol mobilization from liver increases circulating NEFA and induces hepatic *Fgf21* expression

**Figure 2:**
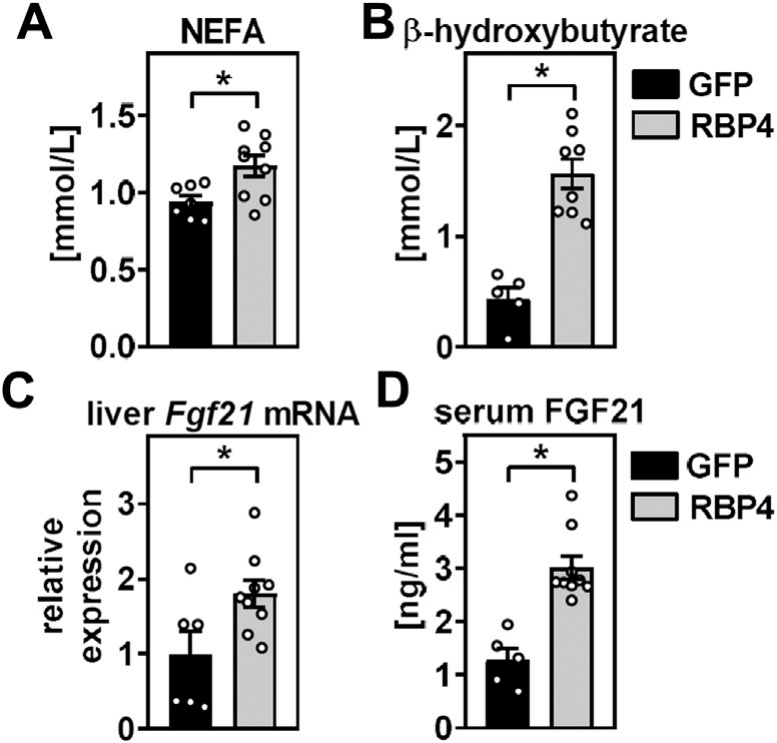
Acute retinol mobilization from liver increases circulating NEFAs and induces hepatic *Fgf21* expression in ad libitum-fed mice. Mice with liver-specific overexpression of GFP or RBP4 were analyzed for (A) NEFAs and (B) β-hydroxybutyrate concentrations in the circulation. C: Expression of *Fgf21* mRNA in liver was determined by quantitative PCR. D: Serum levels of FGF21 were analyzed by ELISA. Data are represented as individual data points and mean ± SEM and ∗*P* < 0.05 versus control mice expressing GFP.

We next determined serum NEFA levels of ad libitum-fed mice. Interestingly, NEFA levels were increased in mice with liver-specific RBP4 overexpression (Fig. 2A). NEFA can be further oxidized in organs like liver and serve as precursors for ketone body synthesis, which we found also increased in the circulation of mice with liver-specific RBP4 overexpression (Fig. 2B), suggesting that hepatic retinol mobilization induces lipolysis and fatty acid oxidation, resulting in increased levels of circulating ketone bodies. We reviewed the literature for liver-derived signals that mediate fat turnover and identified FGF21 as a potential candidate, a fasting-inducible and insulin-sensitizing protein secreted by the liver (36, 37, 38). Interestingly, we found that acute retinol mobilization increased hepatic mRNA expression and serum levels of FGF21 in these mice (Fig. 2C, D).

### Retinol mobilization from primary hepatocytes reduces RARα activity and increases *Fgf21* expression

Circulating FGF21 is primarily liver derived (39), where it is induced by a remarkable diversity of nutritional stressors (40). Earlier studies showed that RARβ overexpression in liver increases (41), whereas a single injection of RA profoundly decreases hepatic expression and circulating concentrations of FGF21 (42), identifying RAR signaling as a regulator of FGF21. We therefore tested whether retinol mobilization by RBP4 affects RAR activity and *Fgf21* expression cell autonomously in hepatocytes.

**Figure 3:**
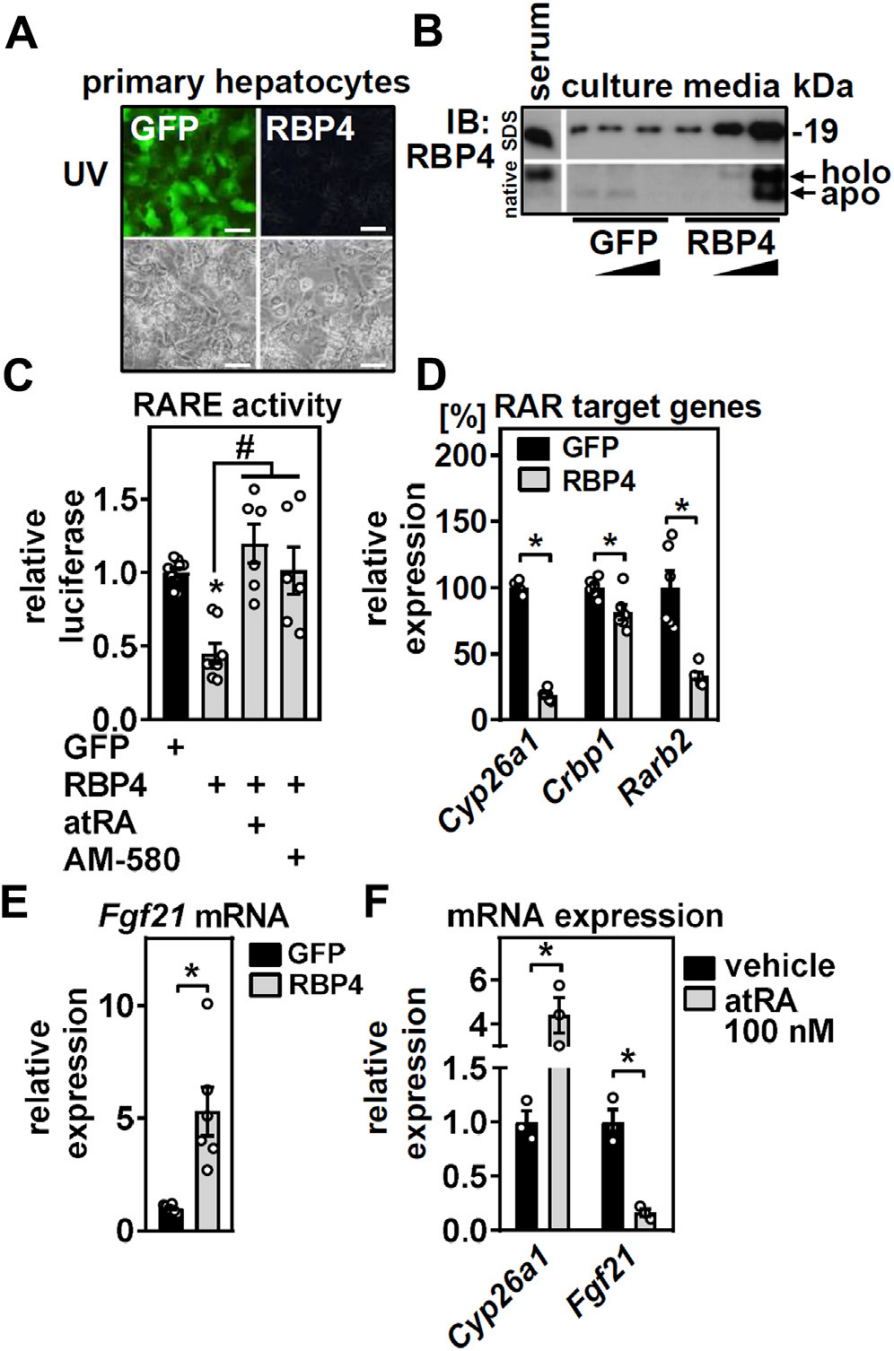
Retinol mobilization from primary hepatocytes reduces RARα activity and increases *Fgf21* expression. Primary mouse hepatocytes were infected with adenoviruses encoding GFP or RBP4 and (A) 48 h later, cells were analyzed by UV and phase contrast microscopy, scale bars represent 100 μm. B: Cell culture media of hepatocytes infected with increasing titers of the indicated adenoviruses were analyzed for total (top) or holo- and apo-RBP4 (bottom) by SDS or native immunoblotting, respectively, whereas mouse serum served as control. C: Primary hepatocytes expressing GFP or RBP4 were transfected with a RARE-driven luciferase reporter and treated with RAR agonists as indicated. Luciferase activity was normalized to activity of coexpressed renilla luciferase. D: Hepatocytes described in (A) were analyzed for the expression of RAR target genes and (E) *Fgf21* by quantitative PCR. F: Primary hepatocytes were incubated with 100 nM of atRA for 24 h, and expression of *Cyp26a1* and *Fgf21* was determined by quantitative PCR. Data are shown as individual data points and mean ± SEM and ∗*P* < 0.05 versus hepatocytes expressing GFP or vehicle treatment and ^#^*P* < 0.05 versus vehicle-treated hepatocytes expressing RBP4.

We infected primary murine hepatocytes with adenoviruses expressing GFP or murine RBP4 (Fig. 3A) and analyzed RBP4 protein in cell culture media. RBP4 overexpression in primary hepatocytes was adenovirus titer dependent and led to a strong increase in total RBP4 in cell culture media whose detection also includes serum-derived bovine RBP4 (Fig. 3B, top panel of SDS-treated samples). Moreover, SDS-free native immunoblotting revealed that hepatocyte-secreted RBP4 accumulated as both retinol-containing holo-RBP4 (upper band) and retinol-free apo-RBP4 (lower band) in media (Fig. 3B, bottom panel of native blot). The increase in holo-RBP4 suggests functional retinol mobilization from primary hepatocytes, which is in accordance with our previous finding that media from RBP4-overexpressing hepatocytes upregulate RAR target genes in undifferentiated 3T3-L1 cells, most likely as a consequence of retinol delivery to these cells by holo-RBP4 (28). Since retinol binding to intracellular RBP4 is considered a prerequisite for RBP4 secretion (43, 44), we would assume that reverse retinol uptake from secreted holo-RBP4 by hepatocytes rather than apo-RBP4 secretion because of depleted retinol stores causes detectable apo-RBP4 in cell culture media of these hepatocytes (Fig. 3B).

Primary hepatocytes were transfected with a luciferase reporter under the control of an RARE and revealed that overexpression of RBP4, using the highest titer shown in Fig. 3B, decreases RARE activity (Fig. 3C). This decrease was rescued by supplementing 500 nM of atRA or by the RARα-selective agonist AM-580 (45) (Fig. 3C), suggesting that retinol mobilization upon RBP4 overexpression reduces RARE activity by limiting the abundance of endogenous RARα-activating ligands such as atRA. Consistently, RBP4 overexpression downregulated canonical RARα target genes, such as cytochrome P450, family 26, subfamily a, polypeptide 1 (*Cyp26a1*), cellular RBP1 (*Crbp1*), and retinoic acid receptor β2 (*Rarb2*) (Fig. 3D). *Fgf21* expression, on the other hand, was increased upon RBP4-induced retinol mobilization (Fig. 3E). To further delineate whether *Fgf21* may indeed behave as a negatively regulated RAR target gene, we exposed primary hepatocytes to 100 nM of atRA and found a robust downregulation of *Fgf21* expression, whereas that of the atRA-inducible target gene *Cyp26a1* showed the expected upregulation (Fig. 3F). Thus, *Fgf21* expression in hepatocytes/liver is negatively regulated by atRA, which is in accordance with previous observations (42). We conclude that retinol mobilization by RBP4 in primary hepatocytes decreases RAR activity and the expression of atRA-inducible target genes but induces expression of *Fgf21*.

### Hepatic FGF21 overexpression induces weight loss and improves systemic metabolism in mice

Next, we explored whether FGF21 may feedback to regulating systemic retinoid homeostasis. We chose to express FGF21 via adeno-associated viruses (AAV), serotype 2/8, driven by a synthetic and highly hepatocyte-specific promoter (27, 29).

**Figure 4:**
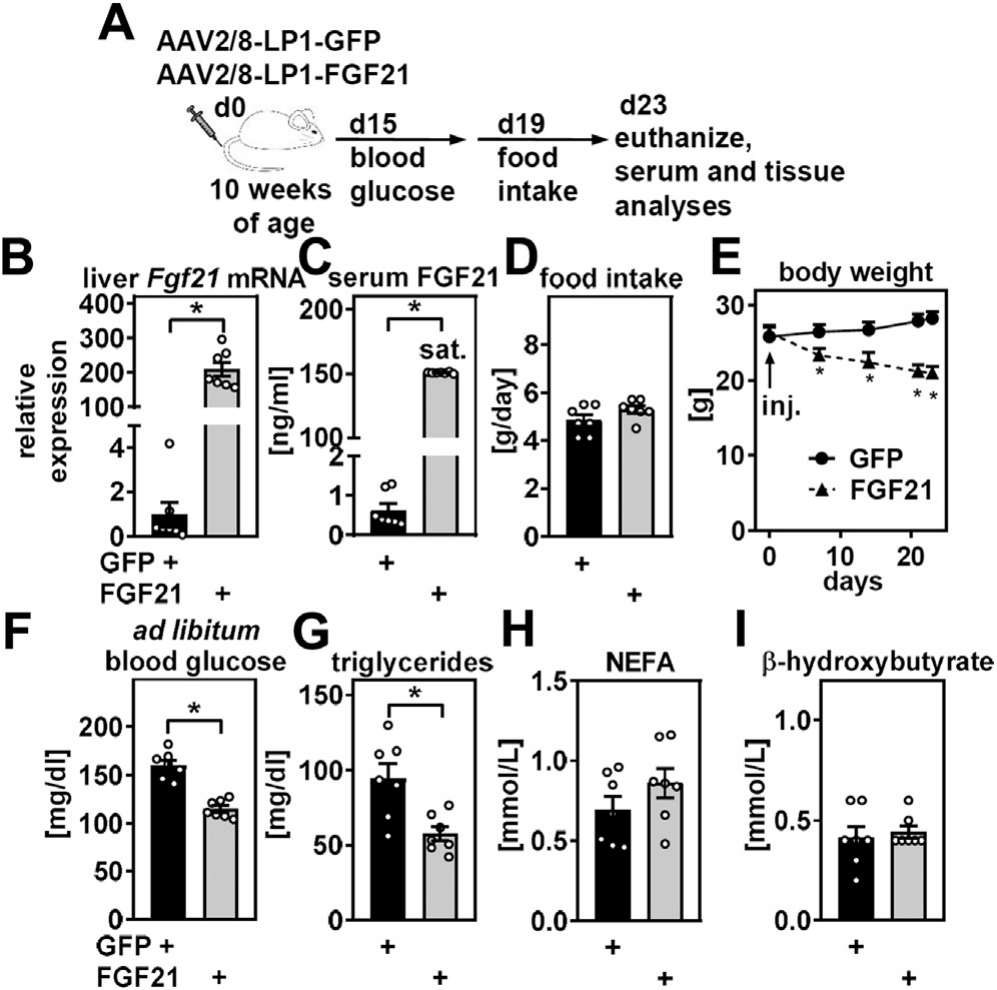
Hepatic FGF21 overexpression induces weight loss and improves systemic metabolism in mice. Male C57BL/6J mice were tail vein-injected with AAV 2/8 to express GFP or FGF21 specifically in liver and characterized as in (A). B: At the end of the study, expression of *Fgf21* in liver was determined by quantitative PCR and (C) serum levels of FGF21 analyzed by ELISA. D: Food intake (24 h) and (E) body weight development of mice with liver-specific overexpression of GFP and FGF21. F: Blood glucose and serum levels of (G) triglycerides, (H) NEFAs, and (I) blood β-hydroxybutyrates were determined at the (A) indicated time points after virus injection. Data are represented as individual data points and/or mean ± SEM and ∗*P* < 0.05 versus control mice expressing GFP.

Mice were tail vein-injected with AAV and characterized as depicted in Fig. 4A. AAV-mediated FGF21 expression led to strongly increased liver mRNA and circulating levels of FGF21 (Fig. 4B, C). Despite several dilution steps, detection of FGF21 by ELISA resulted in saturated absorbance in mice overexpressing FGF21, suggesting that absolute serum concentration may exceed 150 ng/ml in these mice. Food intake trended to be higher but did not reach statistical significance (Fig. 4D). On the other hand, FGF21 expression robustly decreased body weights throughout the study in an organ-specific manner, including a depot-specific reduction in pgWAT but not inguinal WAT (ingWAT) mass (Figs. 4E and S1). FGF21 lowered ad libitum-fed blood glucose and triglyceride levels (Fig. 4F, G), whereas we did not detect alterations in circulating NEFA and β-hydroxybutyrate (Fig. 4H, I). Overall, these findings are in agreement with previous studies (36, 37, 38, 46, 47) and show beneficial metabolic effects of FGF21 in this mouse model.

### FGF21 increases RBP4 and RAR target gene expression in liver

**Figure 5:**
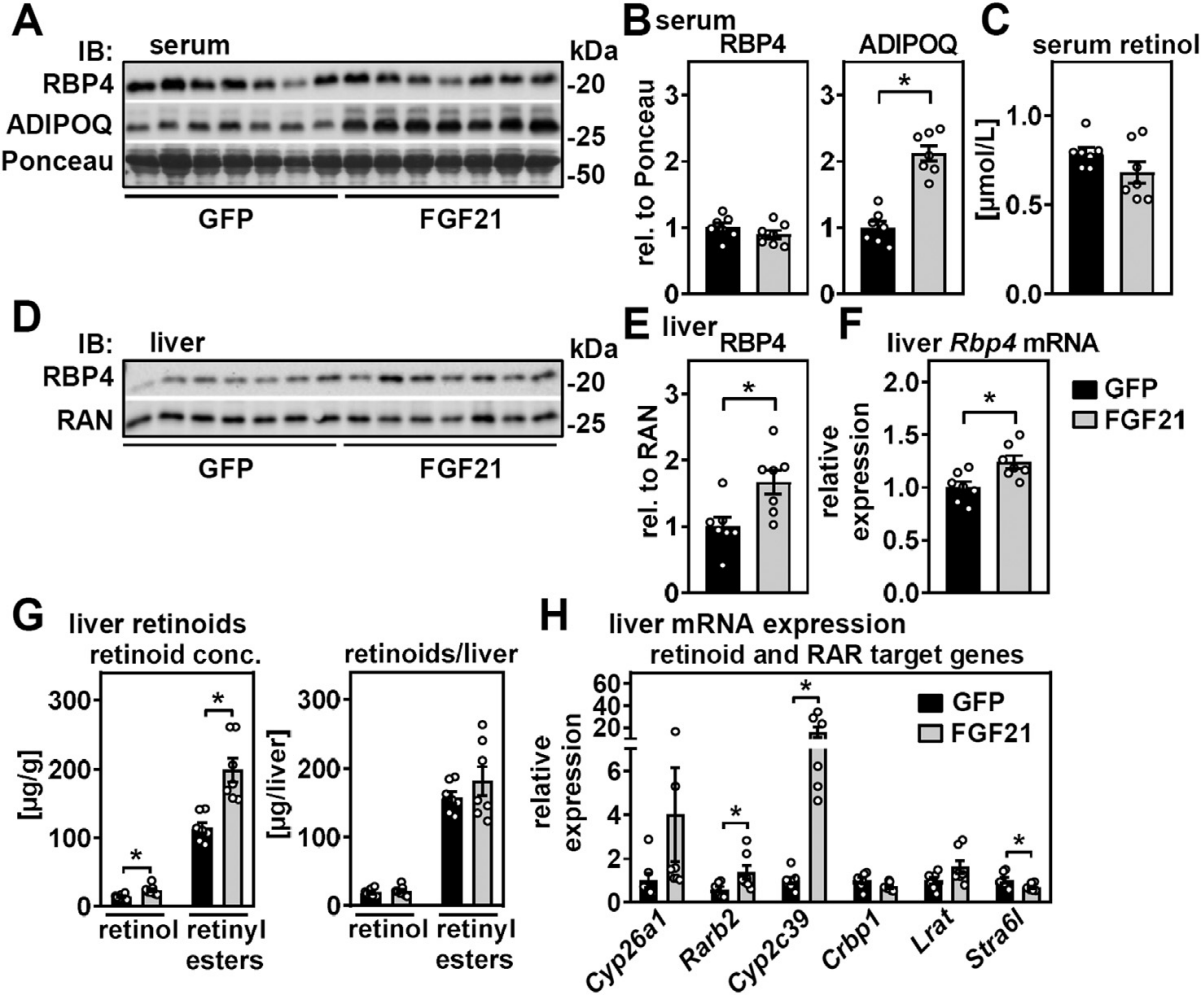
FGF21 increases RBP4 and RAR target gene expression in liver. A: Serum of mice with liver-specific overexpression of GFP and FGF21 was analyzed for the abundance of RBP4 and adiponectin (ADIPOQ) by immunoblotting and (B) evaluated by densitometry. Ponceau staining of the membrane served as loading control. C: Serum retinol was determined by HPLC. D: Hepatic RBP4 protein was analyzed by immunoblotting and (E) evaluated by densitometry. RAN protein expression served as loading control. F: Hepatic mRNA expression of *Rbp4* was determined by quantitative PCR. G: Liver retinol and retinyl esters were quantified by HPLC and expressed relative to tissue mass (left panel) or per total liver (right panel). H: Expression of retinoid-metabolizing and RAR target genes in liver was determined by quantitative PCR. Data are represented as individual data points and mean ± SEM and ∗*P* < 0.05 versus control mice expressing GFP.

Mice with AAV-induced FGF21 expression exhibited increased circulating adiponectin levels, consistent with former reports (48, 49), but serum levels of RBP4 and retinol were unchanged (Fig. 5A–C). In liver, however, both mRNA expression and protein levels of RBP4 were increased (Fig. 5D–F). Similarly, hepatic concentrations of retinol and total retinyl esters were elevated in mice with AAV-mediated expression of FGF21 (Fig. 5G, left panel). Since liver weights were reduced by FGF21 expression (supplemental Fig. S1), hepatic retinoids per whole liver did not differ between the groups (Fig. 5G, right panel). Furthermore, FGF21 overexpression induced several RAR target genes in liver and also expression of *Cyp2c39—*which is a liver-specific and broad-specific CYP enzyme that can metabolize RA, expressed in both hepatocytes and HSC and elevated in livers of RBP4-deficient mice (9, 50, 51). Expression of *Stra6l* (also known as *Rbpr2*), proposed to be negatively regulated by atRA (52), was downregulated (Fig. 5H). We conclude that elevated hepatic FGF21 expression and secretion is associated with higher levels of RBP4 in liver and a hepatic gene expression profile that could be indicative for increased RAR activation and RA catabolism, without affecting steady-state levels of circulating RBP4 and retinol.

### FGF21 induces depot-specific effects on retinoid abundance and RAR target gene expression in adipose tissue

**Figure 6:**
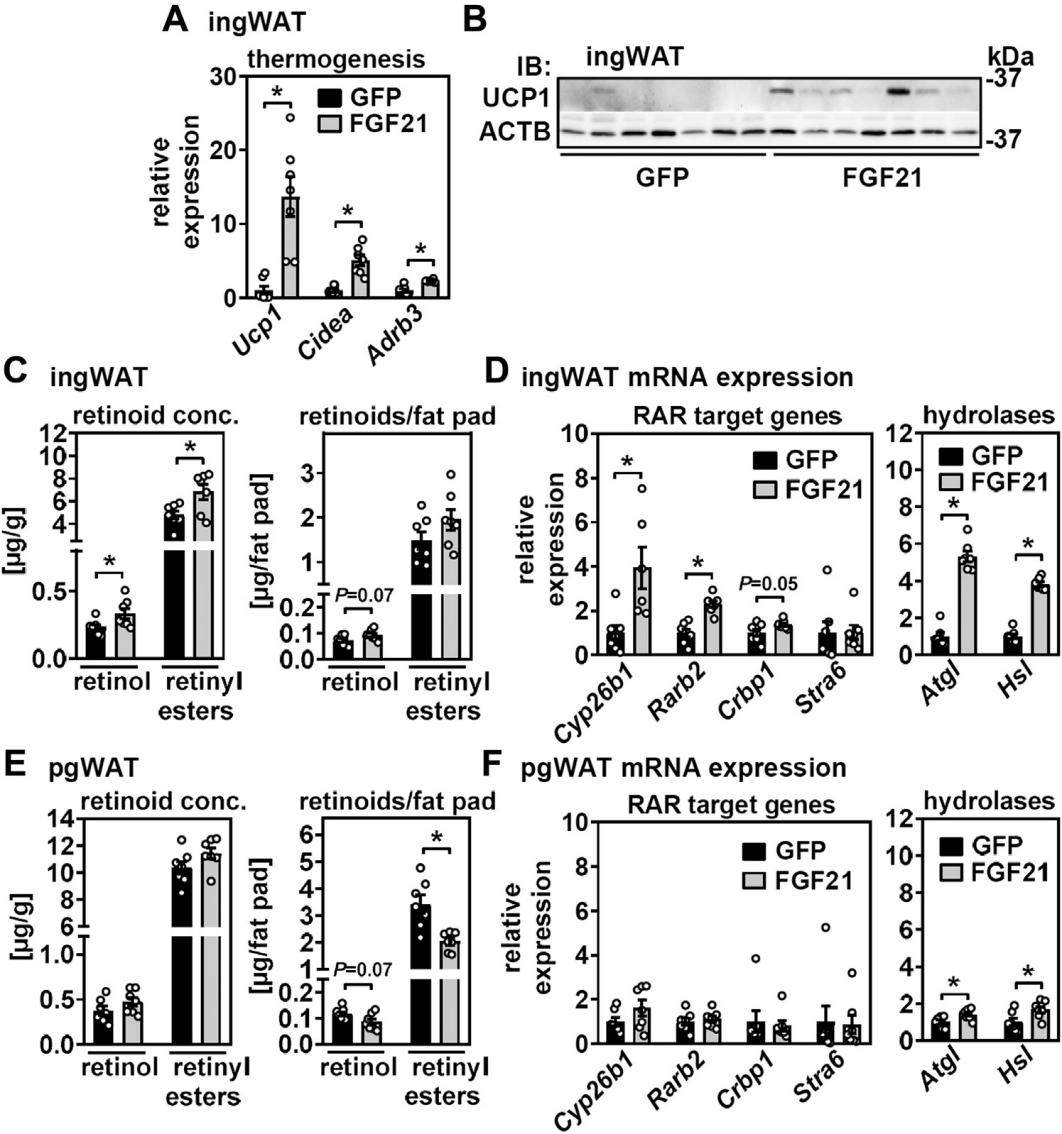
FGF21 induces depot-specific effects on retinoid abundance and RAR target gene expression in adipose tissue. ingWAT of mice with liver-specific overexpression of GFP and FGF21 was analyzed (A) for the expression of thermogenic genes by quantitative PCR (qPCR) and (B) for UCP1 protein by immunoblotting. ACTB served as loading control. C: ingWAT retinol and retinyl esters were quantified by HPLC and expressed relative to tissue mass (left panel) or per total fat pad (right panel). D: Expression of RAR target genes (left panel) and that of hydrolases (right panel) in ingWAT was determined by qPCR. E: pgWAT retinol and retinyl esters were quantified by HPLC and expressed as in (C). F: Expression of RAR target genes (left panel) and that of hydrolases (right panel) in pgWAT was determined by qPCR. Data are represented as individual data points and mean ± SEM and ∗*P* < 0.05 versus control mice expressing GFP.

FGF21 has been shown to induce thermogenic gene expression and browning of WAT in mice, in particular in ingWAT (47, 53). Consistently, elevated circulating FGF21 levels by hepatic overexpression increased mRNA expression of uncoupling protein 1 (*Ucp1*), cell death inducing DFFA like effector a (*Cidea*), and adrenergic receptor, beta 3 (*Adrb3*) in ingWAT (Fig. 6A). UCP1 protein levels in ingWAT, usually hardly detectable in mice that are housed at 21°C, were strongly induced by FGF21 (Fig. 6B). UCP1 protein levels in pgWAT, irrespective of hepatic GFP or FGF21 overexpression, could not be detected (data not shown).

Retinol and total retinyl ester concentrations in ingWAT were increased (Fig. 6C, left panel). After normalization to tissue weights (supplemental Fig. S1), their ingWAT content appeared to be unchanged (Fig. 6C, right panel). Expression of the RAR target genes *Cyp26b1*, *Rarb2*, and *Crbp1* was increased, whereas that of *Stra6*, known to be expressed at very low levels in WAT, did not change (Fig. 6D, left panel). Moreover, mRNA expression of FGF21-inducible adipose triglyceride lipase (*Atgl*) and hormone-sensitive lipase (*Hsl*) (37), both known to also exhibit retinyl ester hydrolase activity (54, 55), was increased (Fig. 6D, right panel). In contrast to ingWAT, hepatic FGF21 overexpression did not increase the concentrations of retinoids in pgWAT and rather reduced total retinoid content of this fat depot (Fig. 6E, left and right panels). Similarly, AAV-mediated FGF21 expression had no effect on RAR target gene expression in pgWAT and only moderately increased *Atgl* and *Hsl* expression (Fig. 6F, left and right panels). Taken together, we found that FGF21 induces browning of ingWAT, coinciding with a maintenance of ingWAT mass, retinoid content, and increased expression of RAR target genes and lipases. In pgWAT, on the other hand, FGF21 induces a loss of tissue mass and retinoid content without inducing RAR signaling.

## Discussion

In this study, we discovered that an acute mobilization of hepatic retinyl esters by forced *Rbp4* expression in mouse liver increases hepatic expression and serum levels of FGF21. This acute retinyl ester mobilization reduced body fat content and lowered serum insulin levels. FGF21 is known to induce very similar effects (40), suggesting that elevated FGF21, at least in part, contributes to the observed metabolic alterations. Notably, long-term overexpression of *Rbp4* in mouse liver for 6 months did not increase hepatic *Fgf21* expression (supplemental Fig. S2) and, somewhat consistently, also lacked effects on serum insulin levels (27). *Rbp4* overexpression in that study was driven by AAV, which, in contrast to fast and robust transient liver expression by adenoviruses, produces a more settle increase in RBP4 that reaches its maximum after approximately 2 weeks and remains stable for months (27). Thus, differences in kinetics and duration of hepatic *Rbp4* overexpression, and thereby retinol mobilization, may be decisive for whether hepatic *Fgf21* expression is induced. Interestingly, although resulting in a comparable elevation of RBP4 and retinol in mouse serum, chronic AAV-mediated RBP4 overexpression in liver also failed to deplete hepatic retinyl esters (27). This implies homeostatic feedbacks to keep hepatic retinyl ester stores, and potentially also *Fgf21* expression, at steady-state levels during prolonged periods of increased mobilization of retinoids from liver. Whether an impaired mobilization of hepatic retinyl esters, as reported for RBP4-deficient mice, affects FGF21 is currently unknown. However, germline deletion of RBP4 (7, 8) may result in homeostatic feedbacks that prevent major changes in hepatic *Fgf21* expression.

Mechanistically, we show that *Fgf21* expression in hepatocytes is upregulated by RBP4-induced retinol mobilization and reduced by RAR activation. Upon viral *Rbp4* overexpression, primary hepatocytes increased holo-RBP4 secretion into the cell culture media, indicative for functional retinol mobilization in vitro. This mobilization suppressed RARE activity, most likely by reducing the availability of precursors for the synthesis of atRA, the most potent endogenous activator of RAR. In contrast to liver tissue, however, cultivated hepatocytes lose their spatial interactions with HSC for bidirectional transfer of retinol for storage/mobilization. Therefore, depletion of retinoids by RBP4-mediated retinol mobilization and the consequential decrease in RARE activity in hepatocytes may be more pronounced in vitro than in functionally intact liver tissue because of lack of retinol influx from HSC to replenish depleted retinoids in hepatocytes. On the other hand, activation of RAR by atRA decreased *Fgf21* expression in primary hepatocytes, corroborating the negative regulation of this gene by RAR activation, which is in accordance with earlier reports (42). One could speculate that acute retinol mobilization from mouse liver induces *Fgf21* expression via a similar mechanism involving reduced RAR activity. However, this needs to be formally tested in mice lacking one or several isoforms of RAR in liver. Alternatively, the observed upregulation of hepatic *Fgf21* expression in vivo may be indirect, since circulating NEFA concentrations, which we found increased upon *Rbp4* overexpression in liver, may lead to the activation of hepatic peroxisome proliferator-activated receptor α (56, 57), a potent inducer of *Fgf21* expression (36, 37).

Our study investigated metabolic consequences of an acute retinol mobilization from liver by a, admittedly, rather artificial intervention of liver-specific RBP4 overexpression in mice. Physiologically, expression of *Rbp4* in mouse liver is constitutively high and not known to be easily deflected in its dynamic, except by severe depletion of hepatic retinoid stores or a low biosynthetic capacity of the liver, which downregulates expression and/or secretion of RBP4 (21). Intriguingly, and representing an exception to this general notion, cold exposure has been reported to increase the circulating concentrations of retinol and RBP4 in both mice and humans, coinciding with elevated expression of *Rbp4* in mouse liver and being indicative for a cold-induced mobilization of hepatic retinoids (58). Impaired mobilization, because of RBP4 deficiency, led to defects in browning of subcutaneous WAT and adaptive thermogenesis in mice (58), suggesting that hepatic retinol mobilization is required for the adaptation to cold. Thus, cold exposure may represent a physiologic context for this model of acute mobilization of hepatic retinoids. Moreover, elevated FGF21, shown here to be a consequence of an acute retinol mobilization, may very well contribute to this adaptation since FGF21 is a potent activator of brown adipose tissue and inducer of WAT browning (40, 53). Further research is needed to address the physiologic interplay between hepatic retinol mobilization by RBP4 and the regulation of FGF21.

Finally, we addressed reciprocal effects of elevated FGF21 on retinoid homeostasis. Liver-specific overexpression of *Fgf21* induced weight loss and lowered blood glucose and triglycerides, as expected. In contrast to our expectations, serum NEFA and β-hydroxybutyrate levels were unchanged, which might be due to the time point of analysis (after 23 days of FGF21 overexpression), when synthesis and release of these metabolites likely match their uptake and consumption in target tissues. Nevertheless, FGF21 increased mRNA expression and protein abundance of RBP4 in liver without corresponding changes in serum levels, suggesting that FGF21 does not further enhance mobilization of hepatic retinyl esters. Rather, concentrations of retinol and retinyl esters in liver strongly increased, and when calculated as micrograms per total liver, showed no difference when compared with control mice. Hepatic expression of RAR target genes and RA-catabolizing enzymes like *Cyp2c39* may point toward enhanced retinoid turnover in liver upon FGF21 overexpression. In WAT, FGF21 induced depot-specific effects with pronounced browning of ingWAT and was accompanied by a conservation of fat mass and retinoid content. Interestingly, this was associated with increased expression of RAR target genes and lipases that exhibit retinyl ester hydrolase activity, implying increased retinoid turnover without reducing their tissue abundance. In pgWAT, on the other hand, both fat mass and retinoid content declined, without major changes in gene expression. Thus, it appears that FGF21 triggers retinoid turnover/RAR activity specifically in the fat depot that undergoes browning. At this point, enhanced delivery of retinol via RBP4 to replenish tissue retinoids in thermogenic ingWAT but not pgWAT is rather speculative but may be supported by the aforementioned report (58).

Our study exhibits certain limitations. These include differences in the metabolic characterization of the analyzed mouse models and the lack of technically challenging atRA quantification, for instance in liver and hepatocytes, to corroborate RARE activity and RAR target gene expression. We also cannot differentiate between metabolic effects induced by elevated FGF21 or by driving RAR signaling in peripheral tissues (28) in our model of acute retinol mobilization. This is an important issue since also RAR activation, at least in mice, results in weight loss and a general improvement of metabolism (59, 60, 61, 62, 63). We also investigated consequences of elevated FGF21 in the context of pharmacological (>100 ng/ml) rather than physiological elevations (<30 ng/ml) (40). Further studies are needed to address these limitations.

## Data availability

All described data are contained within the article.

## Supplemental data

This article contains supplemental data.

## Conflict of interest

The authors declare that they have no conflicts of interest with the contents of this article.
