# Supplementary material for: Acute retinol mobilization by retinol-binding protein 4 in mouse liver induces fibroblast growth factor 21 expression
Source: J Lipid Res. 2022 Aug 27;63(10):100268. doi: 10.1016/j.jlr.2022.100268 (PMC9493389; doi:10.1016/j.jlr.2022.100268)
Supplement: supporting information [file mmc1.pdf]

SUPPLEMENTAL INFORMATION:

Acute retinol mobilization by retinol-binding protein 4 in mouse liver induces fibroblast growth factor 21 expression

Julia S. Steinhoff[1], Carina Wagner[2], Ulrike Taschler[2], Sascha Wulff[1], Marie F. Kiefer[1], Konstantin M. Petricek[1], Sylvia J. Wowro[1], Moritz Oster[1], Roberto E. Flores[1], Na Yang[1], Chen Li[1], Yueming Meng[1], Manuela Sommerfeld[1], Stefan Weger[3], Andrea Henze[4,5], Jens Raila[6], Achim Lass[2,7] and Michael Schupp[1*]

[1]Charité Universitätsmedizin Berlin, corporate member of Freie Universität Berlin and Humboldt-Universität zu Berlin, Institute of Pharmacology, Berlin, Germany

[2]Institute of Molecular Biosciences, NAWI Graz, University of Graz, Graz, Austria

[3]Charité Universitätsmedizin Berlin, corporate member of Freie Universität Berlin and Humboldt-Universität zu Berlin, Institute of Virology, Campus Benjamin Franklin, Berlin, Germany

[4]Martin Luther University Halle-Wittenberg, Institute of Agricultural and Nutritional Sciences, Halle, Germany

[5]Junior Research Group ProAID, Institute of Nutritional Science, University of Potsdam, Nuthetal, Germany

[6]Department of Physiology and Pathophysiology, Institute of Nutritional Science, University of Potsdam, Nuthetal, Germany

[7]BioTechMed-Graz, Graz, Austria

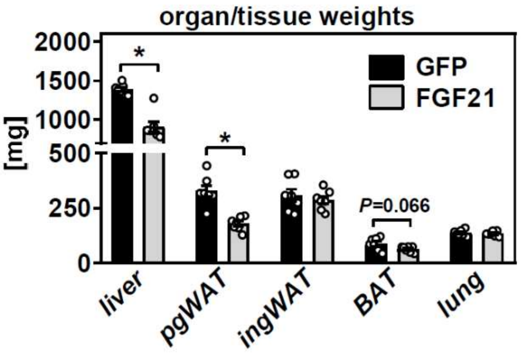

Figure S1. Hepatic FGF21 overexpression affects organ weights. Organ/tissue weights of mice with liver-specific overexpression of GFP and FGF21. Data are represented as individual data points and mean ± sem and *P<0.05 vs. control mice expressing GFP.

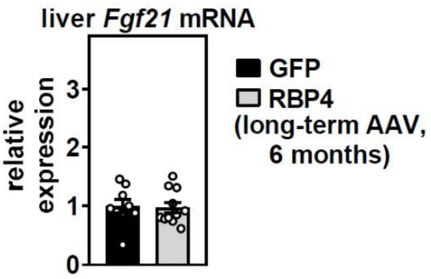

Figure S2. Liver-specific overexpression of RBP4 does not increase hepatic *Fgf21* expression in the long term. Mice were tail vein-injected with adeno-associated viruses expressing GFP or RBP4 and 6 months later analyzed for the expression of *Fgf21* in liver by qPCR. Data are represented as individual data points and mean ± sem.

Supplementary tables

Supplementary Table S1: qPCR primers used in this study (5'-3')

**qPCR**	*GFP* fw	ATACCCTGGTGAATCGCATC
	GFP rv	CATTGTGGGCGTTGTAGTTG
	m36b4 fw	TCATCCAGCAGGTGTTTGACA
	m36b4 rv	GGCACCGAGGCAACAGTT
	mAdrb3 fw	GGCCCTCTCTAGTTCCCAG
	mAdrb3 rv	TAGCCATCAAACCTGTTGAGC
	mAtgl fw	CAACGCCACTCACATCTAC
	mAtgl rv	GTTGAAGGAGGGATGCAGA
	mCidea fw	AGGGACACCACGCATTTCAT
	mCidea rv	CCGATTTCTTTGGTTGCTTG
	mCrbp1 fw	GCGCGCTCGACGTCAAC
	mCrbp1 rv	ACGATCTCTTTGTCTGGCTTCAG
	mCyp26a1 fw	TCTCCAACCTGCACGATTCC
	mCyp26a1 rv	CGGCTGAAGGCCTGCAT
	mCyp26b1 fw	AGAGCAGCAAGGAACATGGC
	mCyp26b1 rv	AGTTGCATGATCAAGGATGTGC
	mCyp2c39 fw	TCCTCTTGAACACGGTCCTC
	mCyp2c39 rv	TAACGGCCTTGGCATTGTTT
	mFgf21 fw	CCTGGGTGTCAAAGCCTCTA
	mFgf21 fw	GTCCTCCAGCAGCAGTTCTC
	mHprt fv	TGCTGACCTGCTGGATTACA
	mHprt rv	TATGTCCCCCGTTGACTGAT
	mHsl fw	CACCCATAGTCAAGAACCCCTTC
	mHsl rv	TCTACCACTTTCAGCGTCACCG
	mLrat fw	TATGGCTCTCGGATCAGTCC
	mLrat rv	TAATCCCAAGACAGCCGAAG
	mRarb2 fv	TGCTCAATCCATCGAGACAC
	mRarb2 rv	CTTGTACACCCGAGGAGGAG

	mRbp4 fw	GCAGGAGGAGCTGTGCCTAGA
	mRbp4 rv	GGAGGGCCTGCTTTGACAGT
	mStra6 fw	GCAGACCAGCTACTCCGAGA
	mStra6 rv	GAGACAGGAGGCTACGCTTG
	mStra6l fw	ACTCCCTGACCTGGTTTGTG
	mStra6l rv	GTGAGGGCCAGTAGAAAACG
	mUcp1 fw	GGGCCCTTGTAAACAACAAA
	mUcp1 rv	ACTGGAGAGGCCAGGAGTGT
**AAV titering**	LP1 fw	GATCCCAGCCAGTGGACTTA
	LP1 rv	GTGCCTCACGACCAACTTCT

Supplementary Table S2: Antibodies used for immunoblotting in this study

Antibody	Species	Product ID	Supplier	Dilution
ACTB	mouse	sc-47778	Santa Cruz Biotechnology	1:1000
ADIPOQ	rabbit	sc-26497	Santa Cruz Biotechnology	1:1000-1:5000
RAN	mouse	610341	BD Biosciences	1:1000-1:2000
RBP4	rabbit	A0040	Dako Denmark A/S	1:500-1:1000
UCP1	rabbit	GTX10983	GeneTex Inc	1:500
